# Unveiling Congenital Heart Disease in an Octogenarian: A Case of Late Diagnosis of Patent Ductus Arteriosus Presenting With Heart Failure With a Preserved Ejection Fraction

**DOI:** 10.7759/cureus.69339

**Published:** 2024-09-13

**Authors:** Muhammad Saleem, Ameer Khan, Abraheem Abraheem, Arbab Hamza

**Affiliations:** 1 Cardiology, Tameside General Hospital, Ashton-under-Lyne, GBR; 2 Cardiology, Tameside Hospital NHS Foundation Trust, Ashton-under-Lyne, GBR

**Keywords:** geriatric patient, heart failure, patent ductus arteriosus, pda, transthoracic echocardiogram

## Abstract

We present the case of an 88-year-old female with a history of chronic obstructive lung disease, atrial fibrillation, and type 2 diabetes mellitus, initially investigated for iron deficiency anemia. Despite inconclusive endoscopic and radiological investigations for occult bleeding, the subsequent evaluation revealed pulmonary hypertension and right heart enlargement on CT of the thorax. Further assessment by echocardiography unveiled dilated pulmonary arteries, right ventricle enlargement, and a left-to-right shunt, consistent with patent ductus arteriosus (PDA), a rare finding in the elderly. Remarkably, the patient recalled being informed of a "weak heart" at birth, suggesting undiagnosed congenital heart disease. Management commenced with heart failure therapy, resulting in significant improvement in dyspnea. This case highlights the importance of considering congenital heart anomalies in elderly patients presenting with heart failure symptoms, emphasizing the value of thorough history-taking and advanced imaging techniques in diagnosis and management.

## Introduction

The ductus arteriosus is the fetal artery connecting the aorta to the pulmonary artery, allowing blood to detour away from the lungs before birth [1]. The normal physiology dictates that the ductus is to spontaneously close within 24-48 hours of birth [1]. However, if the ductus remains open, then this becomes what is known as a patent ductus arteriosus (PDA). PDA accounts for 5%-10% of all congenital heart diseases [1,2]. In the young, PDA is associated with a vast number of complications, including death [1]. The oldest person ever to be diagnosed with PDA was a 92-year-old patient whose diagnosis was made on autopsy [3].

We present the rare case of an 88-year-old patient diagnosed with a PDA. Her diagnosis involved multi-imaging modalities, including numerous transthoracic echocardiograms. The findings were picked on the third transthoracic echocardiogram done over a timeframe of three years.

## Case presentation

An 88-year-old female patient with a past medical history of chronic obstructive lung disease, atrial fibrillation, gastroesophageal reflux disease, and type 2 diabetes mellitus was investigated for iron deficiency anemia in 2022. Subsequently, both endoscopy and colonoscopy were requested, which could not identify any obvious source of her bleeding. A CT of the abdomen and pelvis was requested, which again did not show any evidence of bleeding.

**Figure 1:**
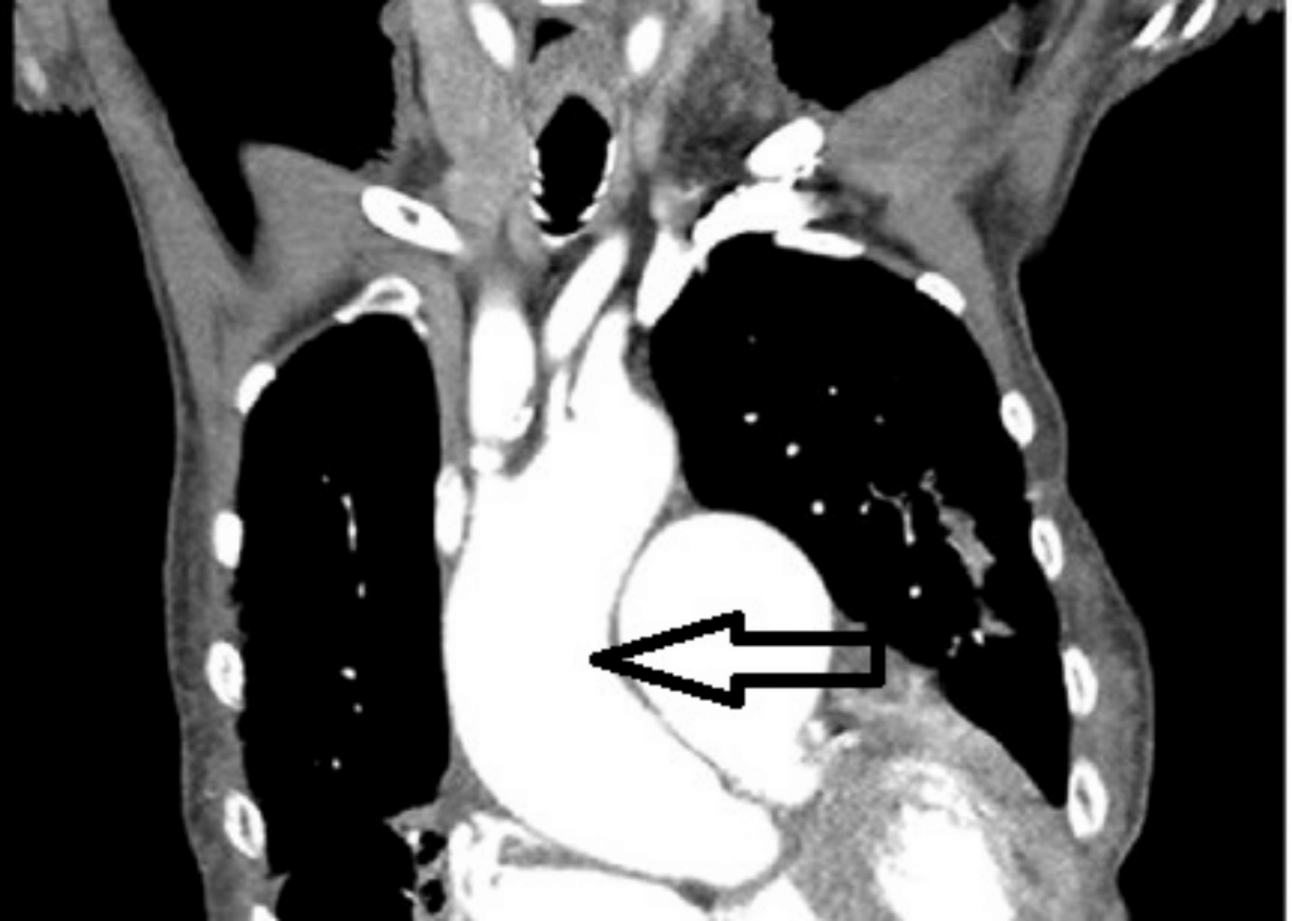
Dilated aortic root on a CT of the thorax (arrow) CT: computed tomography

During her acute admission, her hemoglobin levels (Table 1) improved following blood transfusion. She was then investigated for weight loss, breathlessness, and a mildly raised CA-125 level with a CT of the thorax, abdomen, and pelvis. This showed "cardiomegaly with evidence of right heart chamber's enlargement. Dilatation of main pulmonary arteries including the pulmonary trunk. Multiple lung infiltrates with emphysema" (Figure 1).

**Table 1:** Laboratory blood results MCV: mean corpuscular volume, WCC: white cell count, eGFR: estimate glomerular filtration rate, NT-proBNP: N-terminal prohormone of brain natriuretic peptide, HDL: high-density lipoprotein, LDL: low-density lipoprotein

Laboratory blood results	Result	Normal range
Hemoglobin	111 g/L	115-165 g/L
MCV	85.2 fL	79-95 fL
WCC	3.9×10^9^/L	4-11×10^9^/L
Neutrophils	2.7×10^9^/L	1.8-7.5×10^9^/L
Sodium	146 mmol/L	133-146 mmol/L
Potassium	3.8 mmol/L	3.5-5.3 mmol/L
Creatinine	67 umol/L	54-110 umol/L
Urea	7.4 mmol/L	2.5-7.8 mmol/L
eGFR	79.5 mL/minute	-
NT-proBNP	8,900 pg/mL	<400 pg/mL
Total cholesterol	2.9 mmol/L	<5 mmol/L
Triglycerides	0.7 mmol/L	<1 mmol/L
HDL ratio	1.31	>1.2
LDL ratio	1.3	<4

She was seen by the respiratory physician in outpatient clinics with her worsening dyspnea on the background of her CT scan findings with a high probability of worsening of her pre-existing lung disease. NT-proBNP was requested by the respiratory physician due to the findings of cardiomegaly on the CT scan. The result of NT-proBNP was 8,900 pg/mL. She was referred to the cardiology department for an outpatient evaluation.

An echocardiogram was requested prior to being seen in the cardiology clinic with the elevated NT-proBNP, as her CT of the thorax findings did not completely explain her symptoms of dyspnea.

**Figure 2:**
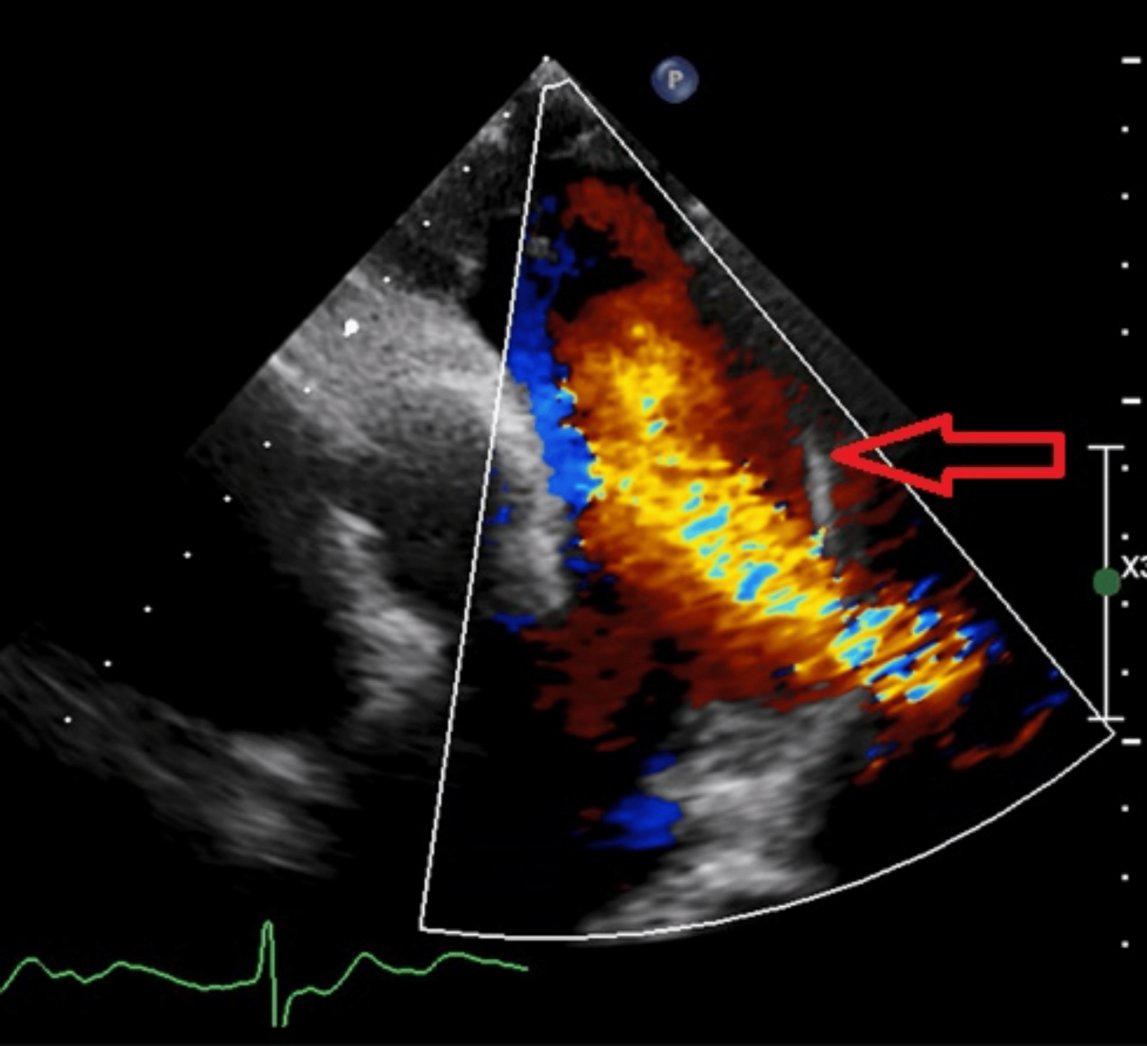
Transthoracic echocardiogram showing a PDA (arrow) PDA: patent ductus arteriosus

A transthoracic echocardiogram (Figure 2) performed in early 2023 showed "dilated pulmonary arteries and right ventricle with impaired systolic function. High probability of pulmonary hypertension. Borderline low left ventricular systolic function of 50%-54%. A continuous flow was detected in the pulmonary artery and in the proximal descending thoracic aorta demonstrating a left to right shunt." These features correlated with a diagnosis of a patent ductus arteriosus.

**Figure 3:**
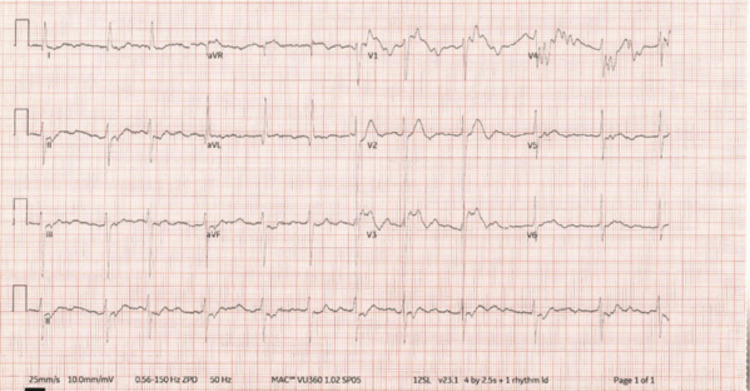
ECG showing rate-controlled AF with signs of LVH ECG: electrocardiogram, AF: atrial fibrillation, LVH: left ventricular hypertrophy

She was followed up in the cardiology outpatient clinics with her echocardiogram findings and a chief complaint of dyspnea. On examination, her chest was clear with good air entry. Her observations were satisfactory. She reported no orthopnea, peripheral edema, or paroxysmal nocturnal dyspnea. Her jugular venous pressure was normal. Her electrocardiogram (ECG) (Figure 3) showed atrial fibrillation that was rate-controlled and signs of left ventricular hypertrophy. She reported that she was a social smoker and drank minimal amounts of alcohol. Her exercise tolerance on flat land allowed her to walk approximately 150 yards with her walking aid before becoming breathless. The two previous transthoracic echocardiograms from 2019 and 2020 did not have any indication of PDA.

This was the first time she was reviewed by a cardiology doctor with evidence supportive of congenital heart disease. On further exploration of the symptoms and past medical history in the clinic, she mentioned that she was told she had a "weak heart" at birth. No corrective surgery or treatment was offered for it nor did she have any pre-existing diagnosis of congenital heart disease.

Based on her presentation and imaging, her diagnosis fits with congestive cardiac failure with a preserved ejection fraction. Based on the New York Heart Association (NYHA) classification of heart failure, she was graded at NYHA 1.

Therefore, medical therapy for heart failure with preserved ejection fraction was started. Her breathlessness markedly improved after the introduction of a gentle dose of diuretic.

Differential diagnosis

Shortness of breath can have a variety of differentials. This patient's iron deficiency anemia can be listed as one. Cardiac differentials would include congestive heart failure and cor pulmonale. The respiratory causes for this patient's shortness of breath could include worsening of the patient's chronic obstructive pulmonary disease. The patient was followed up by the general cardiology team and heart failure nurses for further evaluation and management. Her symptoms remained well controlled on her medication regimen.

## Discussion

A diagnosis of a PDA in an octogenarian is a rarity and highlights the importance of keeping an open mind to have that index of clinical suspicion. PDAs are the most common cardiovascular condition among preterm infants [1,2]. The incidence of silent PDAs, those discovered only by cardiac imaging without clinical manifestations, has been reported to be approximately 5% [1,2]. Untreated PDAs have a reported yearly mortality of up to 1.8% [3].

There is a significant paucity of data on PDAs in this subgroup of the population [4]. As can be seen in the literature, this case would account for the third oldest patient to be diagnosed with a PDA. With the current life expectancy trend expected to increase, it is vital that management strategies are put into practice to aid in the diagnosis and management of late-presenting congenital heart conditions [5].

This case also highlights the impact of diagnostic variability that exists between clinicians. Previous transthoracic echocardiograms had failed to detect this abnormality and showed how the user-to-user variability that exists can impact the detection of conditions such as this.

Echocardiography is the primary imaging modality to diagnose PDAs. The British Society of Echocardiography (BSE) has no formal criteria to aid in the diagnosis of PDAs [3,6]. The American Heart Association has shown that color Doppler can be a sensitive modality in detecting a PDA [1]. A short-axis view with color Doppler all the way through to the pulmonary artery has a greater diagnostic value in capturing defects such as PDAs [7]. Similarly, the American College of Cardiology (ACC) has produced a clinical practice algorithm for a uniform approach to PDAs in the different population age groups [8]. Studies have also shown that in older age groups, CT imaging helps allow for comprehensive evaluation of thoracic structures, thus identifying any significant abnormalities linked to the PDA [2].This is of significance when treatment options are to be considered, especially in the instance of surgery [2].

## Conclusions

Late presentations of congenital heart disease should exist in clinicians' differential diagnoses when investigating patients with shortness of breath. A variety of imaging modalities can help aid management of congenital heart disease in older adults. User-to-user variability exists in reporting of scans; hence, a multidisciplinary team (MDT) approach to such cases could help in achieving early diagnoses. A thorough color Doppler assessment of the aortic and pulmonary vessels can help in the early diagnosis of congenital heart defects.
